# Glecirasib, a Potent and Selective Covalent KRAS G12C Inhibitor Exhibiting Synergism with Cetuximab or SHP2 Inhibitor JAB-3312

**DOI:** 10.1158/2767-9764.CRC-25-0001

**Published:** 2025-05-14

**Authors:** Peng Wang, Xin Sun, Xueting He, Di Kang, Xiaoyu Liu, Dan Liu, Amin Li, Guiqun Yang, Yiwei Lin, Sujing Li, Yinxiang Wang, Yanping Wang

**Affiliations:** 1 Jacobio Pharmaceuticals Co., Ltd., Beijing, China.; 2 Jacobio (US) Pharmaceuticals, Inc., Burlington, Massachusetts.; 3 Jacobio Pharmaceuticals Group Co., Ltd., Beijing, China.

## Abstract

Clinical studies have demonstrated the antitumor efficacy of covalent KRAS G12C inhibitors in treating advanced/metastatic cancers. In the current study, we report the preclinical characteristics of a specific KRAS p.G12C covalent inhibitor, glecirasib. Glecirasib exhibited high potency against KRAS G12C, along with a high level of selectivity over wild-type KRAS, HRAS, and NRAS in biochemical assays. On the cellular level, it substantially reduced downstream ERK and AKT phosphorylation, inhibited the viability of cancer cells harboring the KRAS p.G12C mutation, and demonstrated high selectivity over non-KRAS p.G12C cancer cells. Glecirasib could effectively inhibit HRAS G12C, NRAS G12C, and several G12C-inclusive KRAS double mutants that showed resistance to adagrasib. *In vivo* research suggested that once-daily dosing of glecirasib can robustly inhibit ERK phosphorylation for at least 24 hours and induced tumor regression in several xenograft models, including the NCI-H1373–luciferase intracranial model. Glecirasib in combination with cetuximab or JAB-3312 (sitneprotafib, a clinical-stage SHP2 inhibitor developed by Jacobio Pharma) significantly enhanced antitumor activity both *in vitro* and *in vivo*. Collectively, these results suggest that glecirasib is a potent and selective covalent inhibitor of KRAS G12C, shows potent antitumor activity as monotherapy, and synergizes with either EGFR blockade or SHP2 inhibition. A new drug application for glecirasib has been submitted in China, seeking approval for the treatment of non–small cell lung cancer, supported by a pivotal phase II single-arm study (NCT05009329). Additionally, glecirasib is being explored in clinical trials in combination with cetuximab (phase II, NCT05194995) and JAB-3312 (phase III, NCT06416410).

**Significance:**

Glecirasib potently and selectively inhibits KRAS G12C and reduces ERK and AKT phosphorylation in KRAS G12C–mutant cancer cells, further inducing cell-cycle arrest and apoptosis. Glecirasib monotherapy leads to tumor regression in KRAS G12C–mutant animal models and shows synergistic effects with cetuximab or JAB-3312 (sitneprotafib).

## Introduction

Lung cancer remains the foremost cause of cancer-related mortality globally (1), with non–small cell lung cancer (NSCLC) constituting approximately 85% of lung cancer cases (2). Alterations in various genes, including KRAS, have been implicated in the pathogenesis of NSCLC (3). The treatment of KRAS p.G12C–mutant NSCLC primarily involves chemotherapy and immunotherapy. However, the identification of a targetable regulatory pocket within the KRAS G12C protein has led to the development of specific KRAS G12C inhibitors (4).

Currently, two principal approaches are utilized to design small-molecule KRAS G12C inhibitors. The first strategy involves small molecules that bind directly to GDP-bound KRAS G12C, thereby stabilizing the protein in its inactive state and exerting an anticancer effect (5). This mechanism has led to the approval of four KRAS G12C (OFF) inhibitors. Sotorasib (6–9) and adagrasib (10, 11) were first approved in the United States, whereas IBI351 (12) and garsorasib (13) were recently approved in China. Additional candidates such as divarasib (14), olomorasib, and glecirasib (15) are undergoing clinical evaluation. The second strategy targets KRAS G12C in its active, GTP-bound state. The KRAS G12C (ON) inhibitor RMC-6291 operates by disrupting the interaction between GTP-bound KRAS G12C and its effectors through the formation of a “KRAS: cyclophilin A: drug” tricomplex, thus inhibiting oncogenic signaling (16). RMC-6291 is also under clinical investigation (17).

Glecirasib (JAB-21822) is a potent and selective covalent inhibitor targeting GDP-bound KRAS p.G12C and has shown impressive efficacy and tolerability in pivotal clinical trials in patients with KRAS p.G12C–mutated NSCLC (NCT06008288). Its development was driven by the optimization of a 1,8-naphthyridine-3-carbonitrile scaffold, guided by cocrystal structures and biopharmaceutical modifications to enhance solubility, block metabolic hotspots, and achieve high potency (18). We report here the preclinical evaluation of glecirasib, which efficiently inhibited the growth of a panel of KRAS p.G12C–mutated cancer cells by inducing cell-cycle arrest and apoptosis and showed favorable selectivity against wild-type (WT) KRAS. *In vivo* studies on glecirasib further demonstrated a strong exposure–response relationship and its robust antitumor activities, either as a single agent or in combination with the anti-EGFR antibody cetuximab or the SHP2 inhibitor JAB-3312 (sitneprotafib; ref. 19).

## Materials and Methods

### Cell lines

Capan-2 (RRID: CVCL_0026), LS513 (RRID: CVCL_1386), NCI-H1373 (RRID: CVCL_1465), NCI-H1792 (RRID: CVCL_1495), NCI-H358 (RRID: CVCL_1559), SW1463 (RRID: CVCL_1718), SW1573 (RRID: CVCL_1720), and SW837 (RRID: CVCL_1729) cell lines were obtained from the ATCC). The MIA PaCa-2 (RRID: CVCL_0428) cell line was kindly provided by the Cell Bank of the Chinese Academy of Sciences. The MKN1 (RRID: CVCL_1415) cell line was obtained from Nanjing Cobioer Biosciences. All cell lines were maintained in the medium suggested by the suppliers. *Mycoplasma* contamination was assessed using MycoAlert Detection Kit (Lonza Bioscience). Cell authentication was conducted through short tandem repeat analysis, performed either by the suppliers or a contract research organization. Cells within passages 2 to 20 after thawing were utilized for all experiments.

### Compounds

Glecirasib and JAB-3312 were synthesized by Jacobio Pharma as described in (18) and (19), respectively. Sotorasib and adagrasib were purchased from Beijing Ouhe Technology Co., Ltd. Cetuximab (Erbitux) was produced by Merck Healthcare KGaA.

### Proteins

FLAG-tagged SOS1 ExD (aa 564–1049), His-tagged KRAS (aa 1–169), His-tagged HRAS WT (aa 1–166), and His-tagged NRAS WT (aa 1–172) proteins were all expressed and purified in *Escherichia coli*. GDP and guanosine-5′-[(β,γ)-imido]triphosphate (GppNp) were loaded on RAS by the previously described method (20), and nucleotide loading status was confirmed by ultra-performance liquid chromatography. GST-tagged cRAF RBD (aa 50–132) was purchased from Creative BioMart.

### SOS1-mediated guanine nucleotide exchange of GDP-loaded RAS

The inhibitory activities of compounds on inactive RAS were evaluated by SOS1-mediated guanine nucleotide exchange assays. GDP-loaded KRAS G12D, KRAS G12V, KRAS G12C, KRAS WT, HRAS WT, and NRAS WT proteins were used in this assay. Briefly, GDP-loaded RAS (His-tag) was preincubated with different concentrations of compounds in the presence of 10 nmol/L GDP in a 384-well plate (Greiner) for 1 hour, and then purified SOS1 ExD (FLAG-tag), BODIPY FL GTP (Invitrogen), and mAb anti-6HIS-Tb cryptate Gold (Cisbio/Revvity, RRID: AB_2716834) were added to the assay wells and incubated for another 4 hours at 25°C. Wells containing the same percentage of DMSO served as a vehicle control, and wells without RAS protein served as a low control. Time-resolved fluorescence resonance energy transfer (TR-FRET) signals were read on the Tecan Spark multimode microplate reader (RRID: SCR_021897). The parameters are as follows: F486: excitation 340 nm, emission 486 nm, lag time 100 μs, and integration time 200 μs and F515: excitation 340 nm, emission 515 nm, lag time 100 μs, and integration time 200 μs. TR-FRET ratios for each individual wells were calculated by the following equation: TR-FRET ratio = (signal F515/signal F486) × 10,000. The percentage of activation of compound-treated wells was normalized between the vehicle control and low control [% activation = (TR-FRET ratio_Compound treated_ − TR-FRET ratio_Low control_)/(TR-FRET ratio_Vehicle control_ − TR-FRET ratio_Low control_) ×100%]. The IC_50_ values were calculated using GraphPad Prism (RRID: SCR_002798) by fitting a four-parameter dose–response curve. Details are shown in Supplementary Table S1.

### GppNp-loaded RAS and cRAF interaction assay

The inhibitory activities of compounds on active RAS were evaluated by GppNp-loaded RAS and cRAF interaction assays. GppNp is an analog of GTP. GppNp-loaded KRAS G12D, KRAS G12V, KRAS G12C, KRAS WT, HRAS WT, and NRAS WT proteins were used in this assay.

Briefly, GppNp-loaded RAS (His-tag) was preincubated with different concentrations of compounds in the presence of 200 μmol/L GTP in a 384-well plate for 1 hour, and then cRAF RBD (GST-tag), mAb anti-GST-d2 (Cisbio/Revvity, RRID: AB_2716833), and mAb anti-6HIS-Tb cryptate Gold (Cisbio/Revvity, RRID: AB_2716834) were added to the assay wells and incubated for 2 hours at 25°C. Wells containing same percentage of DMSO served as a vehicle control, and wells without RAS protein served as a low control. Homogeneous time-resolved fluorescence (HTRF) signals were read on the Tecan Spark multimode microplate reader (RRID: SCR_021897), and HTRF ratios were calculated according to the manufacturer’s instructions. The percentage of activation of compound-treated wells were normalized between the vehicle control and low control [% activation = (HTRF ratio_Compound treated_ − HTRF ratio_Low control_)/(HTRF ratio_Vehicle control_ − HTRF ratio_Low control_) ×100%]. The IC_50_ values were calculated using GraphPad Prism (RRID: SCR_002798) by fitting a four-parameter dose–response curve. Details are shown in Supplementary Table S2.

### K_inact_/K_I_ determination

K_inact_/K_I_ was measured by the similar method described above but without compound preincubation. Briefly, 5 nmol/L GDP-loaded KRAS G12C (His-tag) was incubated with a series concentration of compounds at 25°C in the presence with 5 nmol/L GDP, 80 nmol/L BODIPY FL GTP (Invitrogen), 0.5 μmol/L FLAG-SOS1 ExD, and 52.5 ng/mL mAb anti-6HIS-Tb cryptate Gold (Cisbio/Revvity, RRID: AB_2716834). The TR-FRET signals (F515 and F486) were recorded every 3 minutes during 1 hour incubation by the Tecan Spark multimode microplate reader (RRID: SCR_021897). K_inact_/K_I_ was calculated by the method previously described (21). The onset constant rate k_obs_ of each concentration of compound was determined by fitting an exponential equation: $\left[P\right]=\frac{v_{i}}{k_{obs}}\left[1-\exp\left(-k_{obs}t\right)\right]$, in which t is time and [P] is the TR-FRET ratio. Then K_inact_ and K_I_ were determined by fitting to a hyperbolic equation $k_{obs}=\frac{K_{inact}\left[I\right]}{K_{I}+\left[I\right]}$, in which [I] is the concentration of the compound.

### Phospho-ERK/ phospho-AKT cellular assays

For phospho-ERK (p-ERK) assays, cells were seeded in 96-well plates with appropriate density (refer to Supplementary Table S3) and incubated overnight, followed by treatment with a series of concentrations of compounds in the assay medium (containing 0.1% FBS) for 2 hours. Then p-ERK1/2 (Thr202/Tyr204) was detected using the advanced p-ERK1/2 (Thr202/Tyr204) cellular kit (Cisbio/Revvity) according to the manufacturer’s instructions. For phospho-AKT (p-AKT) assays, cells with appropriate density (refer to Supplementary Table S3) were treated with a series of concentrations of compound in culture medium for 24 hours in 96-well plates. Then, p-AKT1/2/3 (Ser473) was detected using the p-AKT1/2/3 (Ser473) cellular kit (Cisbio/Revvity) according to the manufacturer’s instructions. HTRF signals were recorded by the Tecan Spark multimode microplate reader (RRID: SCR_021897). The IC_50_ values were calculated using GraphPad Prism (RRID: SCR_002798) by fitting a four-parameter dose–response curve.

Combination indexes (CI) were calculated using the Chou–Talalay method (22). The CI for a 50% effect (CI_50_) = combo(A)/IC_50_(A) + combo(B)/IC_50_(B). Combo(A) is the concentration of drug A in the combination that induced a 50% effect. Combo(B) is the concentration of drug B in the combination that induced a 50% effect. IC_50_(A) is the IC_50_ value when drug A was administrated as a single agent. IC_50_(B) is the IC_50_ value when drug B was administrated as a single agent. CI_50_ < 0.9 indicates synergistic effect, 0.9 ≤ CI_50_ ≤ 1.1 additive effect, and CI_50_ > 1.1 antagonistic effect.

### Cell viability assay

The inhibitory activity of compounds on Ba/F3-engineered cell line viability was tested by Kyinno Biotechnology. Other cell viability assays on human cancer cell lines were conducted in-house. Cells were seeded in 96-well plates and incubated overnight, followed by treatment with various concentrations of compounds or solvent control for 6 days. Cell viability was detected using either the CellTiter-Glo (CTG) luminescent cell viability assay or the CTG 3D cell viability assay (Promega) for 2D and 3D CTG assays, respectively. For 3D CTG assays, cells were plated in round-bottom ultra-low attachment 96-well plates (Corning 7007). The cell density is indicated in Supplementary Table S3. Luminescence signals were recorded by the Tecan Spark multimode microplate reader (RRID: SCR_021897). The IC_50_ values were calculated using GraphPad Prism (RRID: SCR_002798) by fitting a four-parameter dose–response curve.

The synergistic effect in combination studies was determined using SynergyFinder^+^ R package (RRID: SCR_019318; ref. 23). Loewe score >5 indicates synergistic effect, −5 ≤ Loewe score ≤5 additive effect, and Loewe score < −5 antagonistic effect.

### Cysteine proteome analysis

NCI-H358 cells were treated with either DMSO or 1 μmol/L glecirasib (five independent replicates) for 4 hours. The cysteine proteome analysis was performed by IQ Proteomics. Briefly, collected cell pellets were lysed and treated to label remaining unreacted cysteines. Labeled peptides were enriched by streptavidin agarose and analyzed by nano LC/MS. The adjusted *P* values were calculated according to the Benjamini–Hochberg method using the JMP statistical analysis software. Log_2_ fold changes between the glecirasib-treated group and DMSO controls were taken as the abscissa (*x*-axis), and statistical significance (*P* value) of the difference between the glecirasib-treated group and DMSO controls was taken as the ordinate (*y*-axis).

### Apoptosis assay

NCI-H358 cells (6,000 cells/well) were seeded in 96-well plates and cultured overnight. Cells were treated in triplicate with DMSO control, 1 μmol/L glecirasib, or 1 μmol/L staurosporine (MCE) for 2 to 72 hours. Apoptosis was quantified using the Caspase-Glo 3/7 assay system (Promega), following the manufacturer’s instructions. Luminescence signals were measured by the Tecan Spark multimode microplate reader (RRID: SCR_021897).

### Flow cytometry

NCI-H358 cells were seeded in six-well plates at a density of 1 × 10^6^ cells/well and incubated with glecirasib at 1,000, 300, 100, 30, and 10 nmol/L or DMSO for 24 hours in a cell incubator. Cells were then harvested and fixed using FxCycle PI/RNase Staining Solution (Invitrogen). Cell populations at varying stages of the cell cycle were examined using Miltenyi MACSQuant Analyzer and FlowJo software (RRID: SCR_008520) based on the distribution of DNA content.

### Animal studies

#### Pharmacokinetics/pharmacodynamics study

Female NOD SCID mice (NOD/ShiLtJ-*Prkdc*^*scid*^) were obtained from SPF (Beijing) Biotechnology Co., Ltd. Each female NOD SCID mouse was inoculated subcutaneously in the right flank region with 5 × 10^6^ NCI-H358 cells in PBS containing 50% BD Matrigel (Corning) for tumor development. When the mean tumor volume grew to about 500 to 600 mm^3^, the mice were randomly divided into 15 groups based on their tumor volume, each treatment group contained three mice (the vehicle control group contained four mice), and administered either with vehicle or a single dose of glecirasib at 3, 10, 30, and 100 mg/kg. All treatments were administered by oral gavage. Plasma and tumor samples were collected for pharmacokinetics (PK) and pharmacodynamics (PD) analyses at the desired time points. p-ERK1/2 (Thr202/Tyr204) levels in tumors were determined using the advanced p-ERK1/2 (Thr202/Tyr204) cellular kit (Cisbio/Revvity). The concentration of glecirasib in plasma and tumor was quantified by LC/MS-MS. PK/PD correlation was established by comparing p-ERK levels with drug concentration at each time point.

### Cell line–derived xenograft studies

Female BALB/c nude mice (CAnN.Cg-*Foxn1*^*nu*^/Crl, RRID: IMSR_CRL:194) were obtained from Beijing Vital River Laboratory Animal Technology Co., Ltd. Female BALB/c-nu mice (BALB/cAnSlacNifdc-nu) were obtained from SPF (Beijing) Biotechnology Co., Ltd. Female NCG mice (NOD/ShiLtJGpt-*Prkdc*^*em26Cd52*^*Il2rg*^*em26Cd22*^/Gpt) were obtained from GemPharmatech. Subcutaneous tumors were induced by injecting cells in PBS containing 50% BD Matrigel (Corning) in the right flank of female BALB/c-nu mice (NCI-H1373, 5 × 10^6^ cells), female BALB/c nude mice (MIA PaCa-2, 1 × 10^7^ cells), or female NCG mice (NCI-H358, 5 × 10^6^ cells). Treatment started when the average tumor volume had reached approximately 200 mm^3^. Tumor volume was measured twice weekly by calipers, and body weights were recorded twice weekly.

### Patient-derived xenograft studies

The LUN156 (lung, KRAS p.G12C) patient-derived xenograft (PDX) study was conducted by GenenDesign. CR6243 (colorectal cancer, KRAS p.G12C) and CR6256 (colorectal cancer, KRAS p.G12C) PDX studies were conducted by Crown Bioscience. The abovementioned PDX studies used female BALB/c nude mice. Treatment started when the average tumor volume had reached approximately 200 mm^3^. Tumor volume was measured twice weekly by calipers, and body weights were recorded twice weekly.

Tumor volume was calculated using the following formula: (L × W ^2^) / 2, in which L and W refer to the length and width tumor diameter, respectively. Tumor growth inhibition rates were calculated by TGI% = [1− (V_t_−V_t0_)/(V_c_−V_c0_)] × 100%, in which TGI indicates tumor growth inhibition, V_c_ and V_t_ are the mean tumor volumes of control and treated groups at the end of the study, and V_c0_ and V_t0_ are the mean tumor volume of control and treated groups at the start, respectively. All CDX/PDX mouse studies complied with the guidelines of the Institutional Animal Care and Use Committee at each facility with their approvals.

### Intracranial tumor model

The NCI-H1373-Luc intracranial model study was conducted by Crown Bioscience. Each female BALB/c nude mouse (GemPharmatech, BALB/cNj-*Foxn1*^*nu*^/Gpt, RRID: IMSR_GPT:D000521) was inoculated intracerebrally with 5 × 10^4^ NCI-H1373-Luc cells in the 4 μL PBS solution for tumor development. Mice were randomly grouped on day 14 based on the luminescence in mice head. Treatments were started on the day of grouping. Tumor growth was imaged twice weekly after grouping by bioluminescent imaging (IVIS Spectrum BL, PerkinElmer), and body weights were recorded twice weekly. This study was approved by the Institutional Animal Care and Use Committee of Crown Bioscience prior to execution.

### Statistical analysis

Data were presented as the mean with SD or SEM. Statistical analysis of the difference among multiple groups was performed using a one-way ANOVA followed by multiple comparisons using the least significant difference *post hoc* test (equal variance) or the Games–Howell *post hoc* test (unequal variance) based on the result of homogeneity of variance test. All data were analyzed using SPSS 22.0 software (RRID: SCR_002865). *P* < 0.05 was considered statistically significant.

### 330-kinase panel

This study was conducted by ICE Bioscience (Beijing, China). Glecirasib at 10 μmol/L was screened across each kinase in duplicate wells under a Km concentration of ATP. The list of 330 kinases is shown in Supplementary Table S4.

### Data availability

The data for this study were generated at Jacobio Pharma and multiple contract research organizations. The data generated in this study are available from the corresponding author upon reasonable request.

## Results

### Glecirasib potently and selectively inhibits KRAS G12C *in vitro*

**Figure 1:**
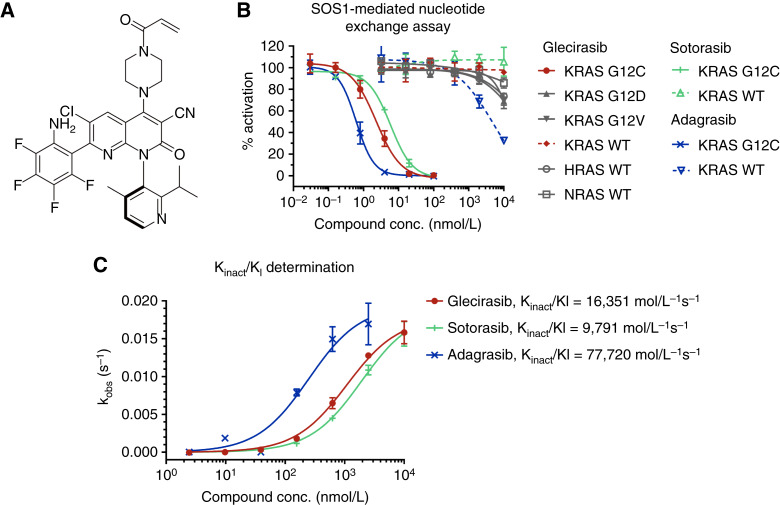
Glecirasib is a potent and selective covalent inhibitor targeting GDP-bound KRAS G12C. **A,** The chemical structure of glecirasib. **B,** Dose–response curves of compounds in SOS1-mediate nucleotide exchange assays (*n* = 2). **C,** The covalent k_inact/_K_I_ second-order rate constant determined by SOS1-mediate nucleotide exchange assay without compound preincubation (*n* = 2). conc., concentration.

Glecirasib, discovered by Jacobio Pharma through structure-based design, is a covalent KRAS G12C inhibitor whose chemical structure is shown in Fig. 1A. Utilizing the SOS1-mediated nucleotide exchange assay, it was shown that glecirasib could potently inhibit GDP-bound KRAS G12C with an IC_50_ value of 2.28 nmol/L and K_inact_/K_I_ of 16,351 M^−1^s^−1^ (Fig. 1B and C). Remarkably, biochemical assays indicated that glecirasib exhibited more than 1,000-fold selectivity on GDP-bound KRAS G12C, as there was a minimal inhibitory effect on GDP-bound KRAS G12D, KRAS G12V, KRAS WT, HRAS WT, and NRAS WT (Fig. 1B; Supplementary Table S5). Additionally, glecirasib did not have inhibitory impacts on GTP-bound RAS, including GTP-bound KRAS G12C, in the GTP-bound RAS and cRAF interaction assays (Supplementary Table S6). Similar to glecirasib, sotorasib and adagrasib also showed no inhibitory activities against GTP-bound KRAS G12C (Supplementary Table S6).

**Figure 2:**
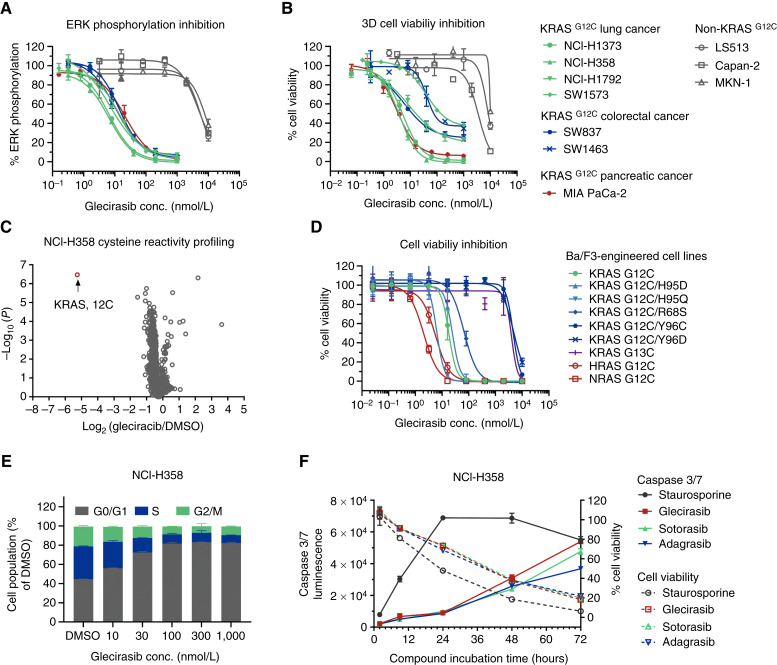
Glecirasib shows high potency and selectivity against cancer cells with RAS G12C mutations *in vitro*, including KRAS G12C, HRAS G12C, NRAS G12C, KRAS G12C/H95, and KRAS G12C/R68S. **A,** Inhibition of p-ERK by glecirasib after 2 hours of treatment on multiple cancer cell lines (*n* = 2). **B,** 3D cell viability inhibition by glecirasib after 6 days of treatment on multiple cancer cell lines (*n* = 2). LS513 harbors KRAS p.G12D, Capan-2 harbors KRAS p.G12V, and MKN1 harbors amplification of WT KRAS. **C,** A profile of NCI-H358 cysteine reactivity with and without glecirasib treatment (1 μmol/L, 4 hours, *n* = 5). **D**, Cell viability inhibition by glecirasib (6 days of treatment) on Ba/F3-engineered cell lines carrying KRAS p.G12C, KRAS p.G12C double mutants, NRAS p.G12C, and HRAS p.G12C (*n* = 3). **E,** Glecirasib induced cell-cycle arrest in NCI-H358 cells, which were treated with glecirasib for 24 hours (*n* = 2). Subsequently, cell populations at varying stages of the cell cycle were examined via flow cytometry. **F,** Time course of caspase 3/7 induction by either glecirasib (1 μmol/L), sotorasib (1 μmol/L), adagrasib (1 μmol/L), or staurosporine (1 μmol/L) in NCI-H358 cells (*n* = 3). conc., concentration.

Given the key role p-ERK plays in downstream signaling pathways mediated by KRAS, p-ERK inhibition assays were conducted. Glecirasib could robustly inhibit ERK phosphorylation in cancer cells carrying KRAS p.G12C, with a median IC_50_ value of 10.9 nmol/L (*n* = 7). By contrast, glecirasib exhibited very weak p-ERK inhibitory activity in non-KRAS p.G12C cancer cells, with a median IC_50_ value of 5,787 nmol/L (*n* = 3; Fig. 2A; Supplementary Table S7). Glecirasib could also potently inhibit cell viability of KRAS p.G12C–carrying cancer cell lines, such as NCI-H1373, NCI-H385, and MIA PaCa-2, with a low median IC_50_ value of 11.8 nmol/L (*n* = 7). In contrast, the median IC_50_ value in cancer cells not carrying this mutation was 8,159 nmol/L (*n* = 3; Fig. 2B; Supplementary Table S7). The abovementioned cell-based studies demonstrated that glecirasib had a remarkable selectivity (>500-fold in both p-ERK and cell viability assays) to specifically inhibit KRAS signaling and the growth of KRAS p.G12C–mutant cancer cells over non-KRAS G12C cells. To further validate the selectivity of the covalent interaction of glecirasib with cellular proteins containing cysteine residues, the cysteine proteome profiling mass spectrometry analysis was performed. Among the 9,378 unique cysteine-containing peptides, the Cys12 peptide in KRAS G12C is the only covalently bound target of glecirasib (Fig. 2C). Taken together, these results indicate that glecirasib is a potent and highly selective inhibitor of GDP-bound KRAS G12C.

In biochemical assays, glecirasib exhibited an IC_50_ value for GDP-KRAS G12C that was lower than that of sotorasib but higher than that of adagrasib (Fig. 1B; Supplementary Table S5). Furthermore, its K_inact_/K_I_ value was higher than that of sotorasib but lower than that of adagrasib (Fig. 1C). These findings suggest that glecirasib has stronger biochemical potency than sotorasib but weaker potency than adagrasib against GDP-KRAS G12C. However, in cellular p-ERK assays, glecirasib demonstrated stronger potency compared with both sotorasib and adagrasib (Supplementary Table S7). Based on our data (Fig. 1B; Supplementary Tables S5–S7), adagrasib exhibited slightly higher off-target activities in KRAS WT biochemical and cellular assays compared with glecirasib and sotorasib. Collectively, these results highlight the unique pharmacologic profile of glecirasib, which combines favorable biochemical and cellular p-ERK activities and high selectivity against KRAS WT.

### Glecirasib potently inhibits HRAS G12C, NRAS G12C, and several G12C-inclusive KRAS double mutants *in vitro*

G12C-inclusive KRAS double mutants, such as G12C/R68S, G12C/H95, and G12C/Y96, were found in patients who were resistant to adagrasib (24). To evaluate the activity of these KRAS mutants, we applied engineered Ba/F3 cell lines with the ectopic expression of G12C-inclusive KRAS double mutants. Glecirasib showed strong inhibition against Ba/F3 cell lines expressing KRAS p.G12C/R68S, KRAS p.G12C/H95D, and KRAS p.G12C/H95Q mutations. In contrast, Ba/F3 cell lines expressing KRAS p.G12C/Y96 mutations were still resistant to glecirasib (Fig. 2D; Supplementary Table S8). These results provided a clue that the His95 mutation in KRAS did not significantly compromise the inhibitory activities of glecirasib. As the major difference found in the switch II pocket is His95 in KRAS but glutamine in HRAS and leucine in NRAS (25), we hypothesized that glecirasib might also inhibit HRAS G12C and NRAS G12C mutations. Indeed, the cell viability inhibitory IC_50_ values for Ba/F3 cell lines carrying KRAS p.G12C, HRAS p.G12C, and NRAS p.G12C mutations by glecirasib were 17.9, 5.32, and 2.02 nmol/L, respectively (Fig. 2D; Supplementary Table S8). Glecirasib showed threefold more potency against NRAS G12C and HRAS G12C compared with KRAS G12C. By contrast, it lacked the inhibitory capacity against KRAS p.G13C–mutant Ba/F3 cells, which further demonstrated high selectivity of glecirasib (Fig. 2D; Supplementary Table S8). Sotorasib was able to inhibit the cell viability of Ba/F3 cell lines harboring NRAS G12C and HRAS G12C, whereas adagrasib showed minimal activity in such cell lines. Consistent with the biochemical and p-ERK assay results, glecirasib exhibited stronger activity than sotorasib in Ba/F3 cell lines with RAS G12C, KRAS G12C/H95X, and G12C/R68S mutations in a head-to-head comparison (Supplementary Table S8).

### Glecirasib inhibits cancer cell viability by inducing cell-cycle arrest and apoptosis

As a key node of the cancer cell signaling pathways, KRAS plays an important role in regulating cell growth, the cell cycle, cell migration, cell survival, etc. (26). In NCI-H358 cells, treatment with glecirasib for 24 hours induced cell-cycle arrest at the G0/G1 phase in a dose-dependent manner (Fig. 2E). In the caspase 3/7 activity assay, glecirasib treatment increasingly induced apoptosis in a time-dependent manner, and the maximal caspase 3/7 activity was reached after 72-hour treatment with glecirasib (Fig. 2F). Although glecirasib exhibited a similar apoptosis-inducing profile to that of sotorasib or adagrasib, it demonstrated slightly stronger apoptotic induction at 72 hours compared with both inhibitors (Fig. 2F). The abovementioned evidence suggests that glecirasib can lead to both cell-cycle arrest and apoptosis on cancer cells with KRAS p.G12C mutations.

### Glecirasib inhibits ERK phosphorylation and growth of KRAS G12C tumors *in vivo*

**Figure 3:**
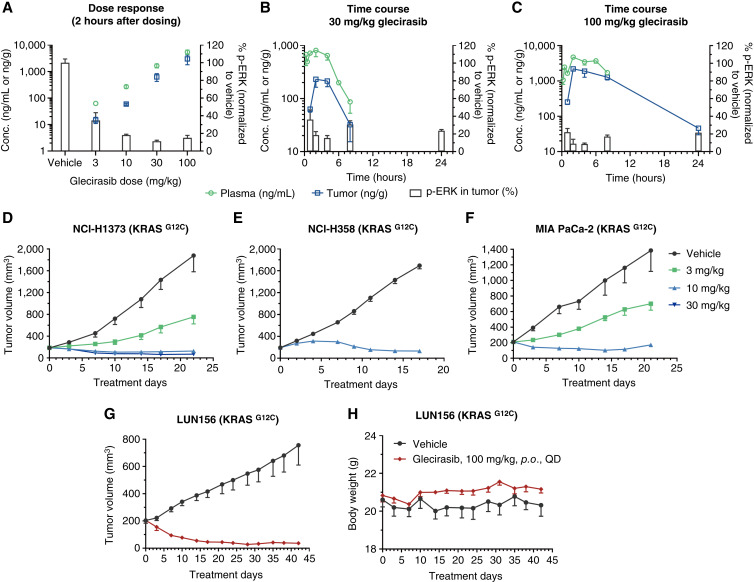
Glecirasib inhibited ERK phosphorylation and growth of tumors carrying the KRAS G12C mutation *in vivo*. **A–C,** PK/PD studies of a single-dose glecirasib administration in NCI-H358 lung cancer xenograft tumors bearing in the flank of mice. Dose response following a single dose of glecirasib is shown in **A**. Time-dependent PK/PD properties after a single dose of 30 mg/kg and 100 mg/kg glecirasib are shown in **B** and **C**, respectively. After 8 hours, glecirasib concentrations in both plasma and tumor in the 30 mg/kg group, as well as the plasma drug concentration in the 100 mg/kg group, were below the quantification limit (plasma: 1 ng/mL; tumor: 5.5 ng/g). Glecirasib concentrations in plasma and tumor tissue were quantified by LC/MS-MS, whereas p-ERK levels in tumors were measured by HTRF. Three mice per group. **D–F,***In vivo* efficacy studies of glecirasib in CDX models: NCI-H1373 lung cancer (**D**), NCI-H358 lung cancer (**E**), and MIA PaCa-2 pancreatic cancer (**F**). NCI-H1373 and MIA PaCa-2 CDX models: six mice per group; NCI-H358 CDX model: eight mice per group. Glecirasib was administrated once a day orally. **G** and **H,** Tumor growth curves (**G**) and mice body weight curves (**H**) of the LUN156 lung cancer PDX model after treatment with glecirasib. Six mice per group. All data are represented as the mean ± SEM. conc., concentration; *p*.*o*., orally; QD, once a day.

In NCI-H358 lung cancer xenograft tumors bearing in the flank of mice, glecirasib inhibited p-ERK in a dose-dependent manner after 2-hour treatment (Fig. 3A). In time-course PK/PD studies, the concentrations of glecirasib in plasma and tumor reached the peak at 2 hours after single oral administration (30 mg/kg and 100 mg/kg), resulting in a maximal p-ERK inhibition in tumors at 2 to 4 hours after treatment. Although the clearance of glecirasib was faster and the concentration in plasma and tumor was already below the lower limit of quantification after 24 hours, a sustained p-ERK inhibition in tumors of more than 70% for at least 24 hours was observed (Fig. 3B and C). This can likely be attributed to the covalent bond formed between glecirasib and KRAS p.G12C along with the long turnover time of the KRAS protein (t_1/2_ ≈ 23 hours; refs. 6, 27).

**Figure 4:**
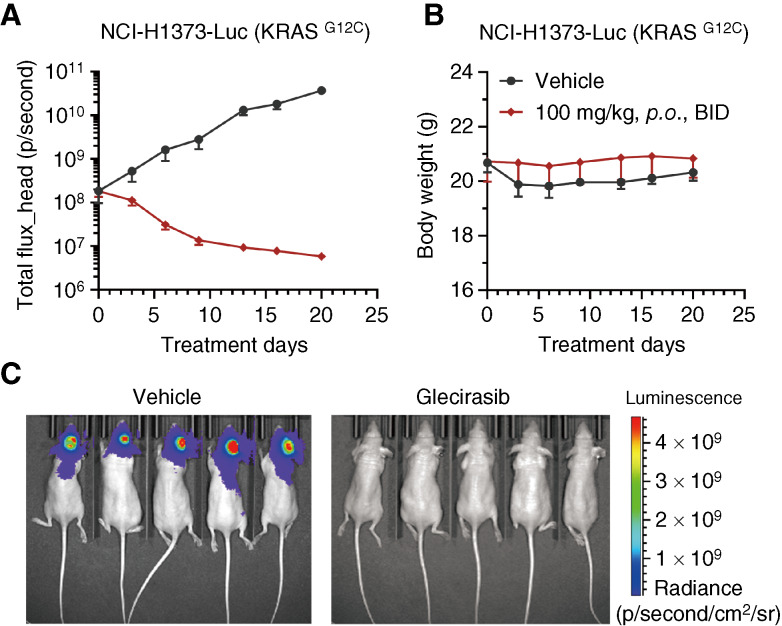
Efficacy of glecirasib in the intracranially implanted NCI-H1373-Luc xenograft model. The curves of bioluminescence in mice head and body weight are shown in **A** and **B**. Data are shown as the mean ± SEM. Images of bioluminescence in individual mouse on day 20 are shown in **C**. Five mice per group. BID, twice a day; *p.o.*, orally.

Further *in vivo* efficacy studies showed that glecirasib dose-dependently inhibited the growth of NCI-H1373 lung cancer and MIA PaCa-2 pancreatic cancer xenograft tumors bearing in the flank of mice, and 10 mg/kg daily dose of glecirasib could induce tumor regression in NCI-H1373, NCI-H358, and MIA PaCa-2 xenograft models (Fig. 3D–F). Glecirasib also showed strong antitumor efficacy in the LUN156 (KRAS p.G12C) lung cancer PDX model. A measure of 100 mg/kg daily dose of glecirasib induced prolonged tumor regression without affecting the body weight of mice (Fig. 3G and H). Considering the high frequency of brain metastases in patients with lung cancer (28), we evaluated the potential efficacy of glecirasib within an intracranial tumor model. In the case of the NCI-H1373-Luc (KRAS p.G12C) model implanted intracranially in mice, a twice-daily dosing regimen was used to enhance intracranial drug exposure. Glecirasib markedly reduced the bioluminescent signals in the brain without inducing significant change in mouse body weight (Fig. 4A and B). In the final bioluminescent images, tumors of all mice treated with glecirasib were below the detection limit of the imaging system (Fig. 4C). Despite the relatively low distribution in the mouse brain (representing 4% of AUC in plasma, as shown in Supplementary Table S9), its potent antitumor activity in the intracranial NCI-H1373-Luc model was nonetheless noteworthy. Furthermore, we observed a slightly higher brain distribution of glecirasib in rats (10% of AUC in plasma), suggesting species-specific differences. Therefore, based on these findings, we propose that even though the brain distribution percentage seems low, the effective concentration of glecirasib can still be achieved in sensitive models—indicating a promising potential for glecirasib’s efficacy in treating lung cancer with brain metastasis.

### Glecirasib in combination with cetuximab synergistically inhibits KRAS G12C

**Figure 5:**
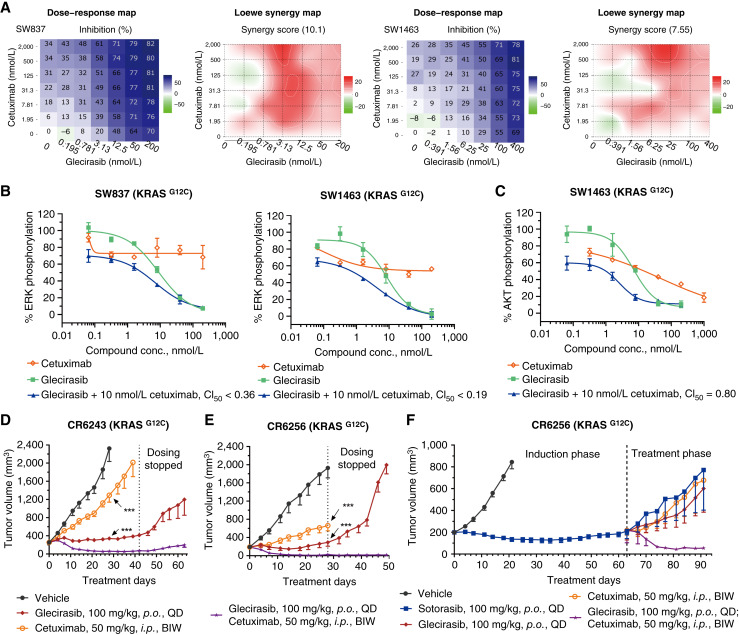
Cetuximab enhanced the antitumor effect of glecirasib in colorectal cancer models. **A,** Cell viability (3D) dose–response map and Loewe synergy map of glecirasib in combination with cetuximab on the indicated cancer cell lines (*n* = 2), with darker blue colors denoting greater cell viability inhibition and deeper red colors demonstrating stronger Loewe synergistic effect. Global Loewe synergy scores are indicated at the top of Loewe synergy maps. Global Loewe synergy score >5 indicates synergistic effect. **B** and **C,** Inhibition of p-ERK (**B**) and p-AKT (**C**) by either glecirasib, cetuximab, or combinations in SW837 and SW1463 cells (*n* = 2). The CI for a 50% effect (CI_50_) was indicated, and CI_50_ < 0.9 indicates synergistic effect. **D** and **E,** Glecirasib and cetuximab combination studies in CR6243 (**D**) and CR6256 (**E**) PDX models. Six mice per group. ***, *P* < 0.001 vs. combination group (day 28). **F,** Mice were treated with 100 mg/kg sotorasib for 63 days and then randomly grouped and treated either with 100 mg/kg sotorasib, 100 mg/kg glecirasib, or 50 mg/kg cetuximab or glecirasib in combination with cetuximab. Three mice per group. All data are represented as the mean ± SEM. BIW, twice a week; conc., concentration; *i.p.*, intraperitoneally; *p*.*o*., orally; QD, once a day.

Despite the noteworthy initial clinical efficacy seen with the use of KRAS G12C inhibitors as a standalone therapy, attention had been drawn to the rapid development of drug resistance, which undermined the therapeutic application (24). When the RAS pathway is suppressed, the upstream receptor tyrosine kinase signaling pathways can be activated via feedback mechanisms, including increased expression of ligands and/or receptors. Clinical findings have indicated that colorectal cancer carrying KRAS mutation generally exhibits a lesser response to KRAS G12C inhibitors compared with NSCLC. One possible reason is that colorectal cancer tends to retain greater basal activation of EGFR (29, 30). As such, we evaluated the combined effects of glecirasib and cetuximab, an EGFR-targeted mAb. Glecirasib plus cetuximab demonstrated significantly synergistic effects in inhibiting cell viability of colorectal cancer cell lines SW1463 and SW837, as evidenced by synergy scores greater than 5 (Fig. 5A). Furthermore, the combination yielded CI_50_ values below 0.9 for both ERK phosphorylation and AKT phosphorylation, further confirming the synergistic inhibition of these signaling pathways in the same cell lines (Fig. 5B and C). Notably, this combined treatment demonstrated enhanced antitumor activity in two PDX models of colorectal cancer, namely CR6243 and CR6256. Specifically, glecirasib in combination with cetuximab induced prolonged and significantly improved tumor regression in both xenograft models. Even after treatment cessation, tumor volume in the combination-treated groups showed slower (CR6243) or negligible (CR6256) rebound compared with the glecirasib monotherapy group during the entire study period (Fig. 5D and E). In comparison, there was evident tumor regrowth observed in the group treated with glecirasib monotherapy despite continuous treatment (Fig. 5E).

To further confirm our findings, we conducted an experiment to evaluate the efficacy of the combination on resistant models which showed tumor rebound following treatment with the KRAS G12C inhibitor. During the induction phase, CR6256 tumors were treated with another KRAS G12C inhibitor, sotorasib. When obvious tumor regrowth was detected, the mice were then randomly assigned to four treatment groups: 100 mg/kg sotorasib, 100 mg/kg glecirasib, 50 mg/kg cetuximab, or combination therapy of glecirasib and cetuximab. It was observed that tumors in the sotorasib group continued to grow, and rebound tumors also exhibited resistance to both glecirasib and cetuximab in monotherapy. By contrast, the combination of glecirasib and cetuximab induced tumor regression (Fig. 5F). These results suggest that the combination of glecirasib and cetuximab may overcome KRAS G12C inhibitor resistance particularly in colorectal cancer.

### Glecirasib in combination with JAB-3312 synergistically inhibits KRAS G12C

Considering the role of SHP2 to activate the RAS pathway through multiple modes of action, including the dephosphorylation of RAS and promoting SOS1 activity (31, 32), there is also strong rationale for the combination of KRAS G12C and SHP2 inhibitors. Therefore, the combination between glecirasib and JAB-3312 (19), a clinical-stage SHP2 inhibitor, was evaluated in a panel of KRAS p.G12C cell lines as well as xenograft models.

**Figure 6:**
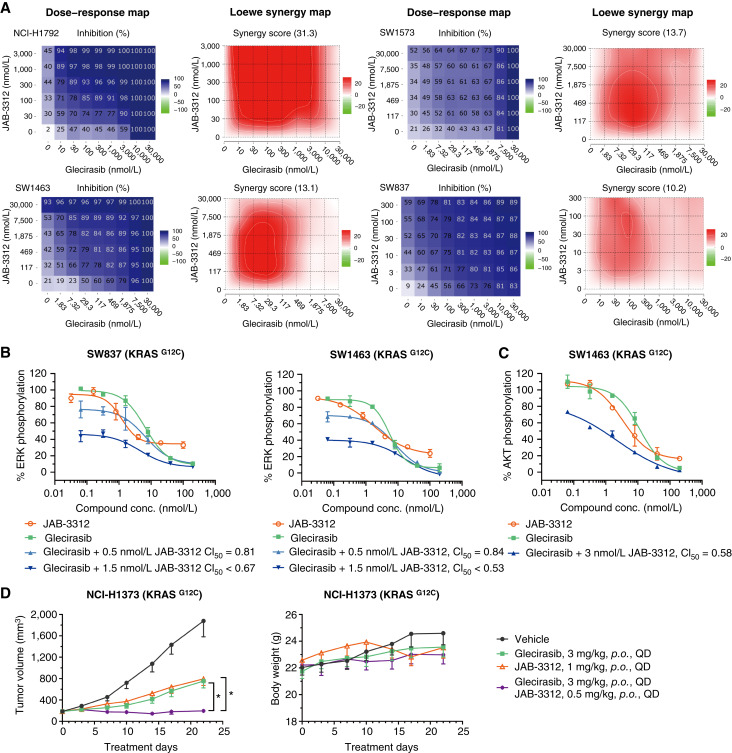
Glecirasib in combination with the SHP2 inhibitor JAB-3312 synergistically inhibited cancer cell growth. **A,** Cell viability (2D) dose–response map and Loewe synergy map of glecirasib in combination with JAB-3312 on the indicated cancer cell lines (*n* = 2), with darker blue colors denoting greater cell viability inhibition and deeper red colors demonstrating stronger Loewe synergistic effect. Global Loewe synergy scores are indicated at the top of Loewe synergy maps. Global Loewe synergy score >5 indicates synergistic effect. **B** and **C,** Inhibition of p-ERK (**B**) and p-AKT (**C**) by either glecirasib, JAB-3312, or combinations in SW837 and SW1463 (*n* = 2). The CI for the 50% effect (CI_50_) was indicated, and CI_50_ < 0.9 indicates synergistic effect. **D,** Glecirasib and JAB-3312 combination studies in the NCI-H1373 lung cancer xenograft model. Six mice per group. *, *P* < 0.05 vs. combination group. All data are shown as the mean ± SEM. conc., concentration; *p*.*o*., orally; QD, once a day.

The combination of glecirasib and JAB-3312 exhibited significantly synergistic effects in inhibiting cell viability across multiple cancer cell lines, including NCI-H1792 (lung cancer), SW1573 (lung cancer), SW1463 (colorectal cancer), and SW837 (colorectal cancer), as demonstrated by synergy scores greater than 5 (Fig. 6A). This combination treatment resulted in CI_50_ values below 0.9 for both ERK phosphorylation and AKT phosphorylation, further confirming the synergistic inhibition of these signaling pathways in SW837 and SW1463 cell lines (Fig. 6B and C). Furthermore, the combined application of glecirasib and JAB-3312 produced significantly higher antitumor efficacy (TGI 99%) compared with either single agent (67% and 64%, respectively) administrated individually in the NCI-H1373 lung cancer xenograft model (Fig. 6D). These findings further demonstrated the potential therapeutic advantage of this combination strategy.

### Glecirasib exhibits a favorable safety profile in preclinical studies

Secondary pharmacology studies suggested that glecirasib had a low risk of off-target effects. Glecirasib did not obviously inhibit a panel of 330 kinases, with PIM2 the only one showing 50% inhibition at 10 μmol/L. All other kinases exhibited an inhibition rate of less than 50% at the same concentration (Supplementary Fig. S1; Supplementary Table S4). In addition, *in vitro* safety panel screening discovered that the only off-target effect for glecirasib at 10 μmol/L was the androgen receptor (Supplementary Table S10), with an IC_50_ value of 3.6 μmol/L. Glecirasib exhibited a favorable safety profile in safety pharmacology and toxicology studies. In good laboratory practice safety pharmacology studies, no risk of QT interval prolongation was observed in conscious beagle dogs (Supplementary Table S11). Glecirasib was also proved to have no adverse effect on both the respiratory and central nervous systems (Supplementary Table S11). In addition, glecirasib was tested negative in the *in vitro* Ames and chromosomal aberration studies, the *in vivo* rat bone marrow micronucleus assay, and the *in vivo* guinea pig phototoxicity study (Supplementary Table S12).

## Discussion

In our report, we detailed the preclinical properties of glecirasib, a potent, selective, covalent inhibitor specific to GDP-bound KRAS G12C. Remarkably, glecirasib exhibited >500-fold selectivity in both biochemical and cellular assays against WT KRAS. Glecirasib potently suppressed the downstream signaling of KRAS, including p-ERK (Fig. 2A) and p-AKT (Figs. 5C and 6C). In addition, even at 10 μmol/L glecirasib, it did not obviously inhibit kinases in MAPK and PI3K signaling pathways in a panel of 330 kinases (Supplementary Fig. S1; Supplementary Table S3), whereas its IC_50_ value on KRAS G12C is 2.28 nmol/L. This evidence confirmed that its inhibitory activities on p-ERK and p-AKT were due to KRAS inhibition rather than off-target inhibition on kinases. Further cysteine–proteome profiling analysis also demonstrated high selectivity of glecirasib.

Beyond KRAS, the findings revealed that glecirasib also exhibited strong inhibitory effects on HRAS G12C and NRAS G12C, which rendered it as a pan-RAS G12C inhibitor. Similarly, the KRAS G12C inhibitors sotorasib and JDQ443 had also displayed inhibitory effects on HRAS G12C and NRAS C12C, with the combination therapy of sotorasib and EGFR antibody panitumumab being successfully used to treat a patient with colorectal cancer with NRAS p.G12C mutation (33). Although these HRAS p.G12C and NRAS p.G12C mutations are relatively rare in patients with cancer, these findings suggest that glecirasib could offer new treatment options for these patient populations. Drug resistance presents a major challenge for the clinical application of KRAS G12C inhibitors. Glecirasib, however, had shown strong inhibitory effects on secondary mutants such as G12C/R68S, G12C/H95D, and G12C/H95Q, which were known to exhibit resistance to adagrasib (24). It is hypothesized that glecirasib could impede the emergence of G12C/H95 and G12C/R68S mutations in patients and perhaps remain effective in patients who developed those mutations after initial adagrasib treatment. Cocrystal structures have revealed that adagrasib formed a vital hydrogen bond with the His95 of KRAS G12C (34), a bond not observed with glecirasib (18), sotorasib (33), or JDQ443 (35). This feature contributes to the differential RAS isoform inhibition profile between glecirasib and adagrasib. Particularly, adagrasib cannot inhibit the KRAS G12C mutation with a secondary His95 mutation, HRAS G12C, or NRAS G12C, as the latter two possess glutamine and leucine at the 95 position, respectively. In contrast, glecirasib demonstrated strong inhibition against KRAS G12C/H95, HRAS G12C, and NRAS G12C mutations.

*In vivo* studies showed that once-daily dosing of glecirasib can strongly inhibit p-ERK for at least 24 hours and induce maximal tumor responses ranging from regression (NCI-H1373, NCI-H358, MIA PaCa-2, and LUN156) to stasis (CR6243 and CR6256). Glecirasib’s notable efficacy in an intracranial tumor model suggests its potential for treating KRAS G12C–driven lung cancer with brain metastasis.

In patients with locally advanced or metastatic KRAS G12C–mutated NSCLC, glecirasib at 800 mg daily resulted in a confirmed overall response rate of 47.9% (56/117), disease control rate of 86.3% (101/117), and median progression-free survival of 8.2 months by an Independent Review Committee in a pivotal phase II single-arm study (NCT05009329; ref. 15). A new drug application for glecirasib has been filed in China for treating patients with locally advanced or metastatic NSCLC who carry the KRAS p.G12C mutation and have received at least one prior systemic treatment. Despite the encouraging initial efficacy of KRAS G12C inhibitors, rapid drug resistance inevitably occurs, and EGFR signaling reactivation is one of the main resistance mechanisms to KRAS G12C inhibitors in patients with colorectal cancer. KRAS inhibitors (e.g., sotorasib and adagrasib) in combination with the EGFR antibody have been proved more effective than either agent alone in patients with colorectal cancer (36, 37). Consistently, combining glecirasib with cetuximab, an EGFR antibody, enhanced antitumor efficiency against colorectal cancer in preclinical models. Glecirasib plus cetuximab is under clinical investigation and was approved in a registrational phase III clinical trial in patients with KRAS G12C–mutated colorectal cancer in China. In addition, SHP2 activates the RAS pathway through the dephosphorylation of RAS and promotion of SOS1 activity (31, 32), suggesting a strong rationale of combining glecirasib with JAB-3312 (19). This combination significantly enhanced the antitumor activity of either single agent in preclinical models. In the phase I/IIa clinical trial, glecirasib plus JAB-3312 showed encouraging results. Further evaluation of this combination in the first-line setting, compared with standard-of-care treatment in NSCLC (i.e., PD-1 antibody plus pemetrexed and carboplatin), is ongoing in a randomized and registrational phase III trial (NCT06416410).

## Supplementary Material

Figure S1 Figure S1 shows glecirasib's kinases profiling tree.

Table S1 Table S1 shows assay conditions of SOS1-mediated guanine nucleotide exchange assays.

Table S2 Table S2 shows assay conditions of GppNp-RAS and cRAF interaction assays.

Table S3 Table S3 shows cell lines information of cellular assays.

Table S4 Table S4 shows kinases list in an order from strong to weak by inhibition rate of 10 μM glecirasib.

Table S5 Table S5 shows IC50 values in SOS1 mediate nucleotide exchange assays.

Table S6 Table S6 shows IC50 values in GppNp-bound RAS and cRAF interaction assays.

Table S7 Table S7 shows IC50 values in p-ERK inhibition assays and cell viability assays.

Table S8 Table S8 shows cell viability IC50 values in Ba/F3 engineered RAS cell lines.

Table S9 Table S9 shows glecirasib's AUC in plasma and brain following a single oral dose.

Table S10 Table S10 shows the data of in vitro safety panel screening.

Table S11 Table S11 shows the summary of glecirasib's in vivo safety pharmacology studies.

Table S12 Table S12 shows the summary of glecirasib's genotoxicity and phototoxicity studies.
