# Supplementary figures and images for: Glecirasib, a Potent and Selective Covalent KRAS G12C Inhibitor Exhibiting Synergism with Cetuximab or SHP2 Inhibitor JAB-3312
Source: Cancer Res Commun. 2025 May 14;5(5):792–803. doi: 10.1158/2767-9764.CRC-25-0001 (PMC12076188; doi:10.1158/2767-9764.CRC-25-0001)

Supplementary Figure 1. 330 kinases profiling of glecirasib (10  $\mu$ M).

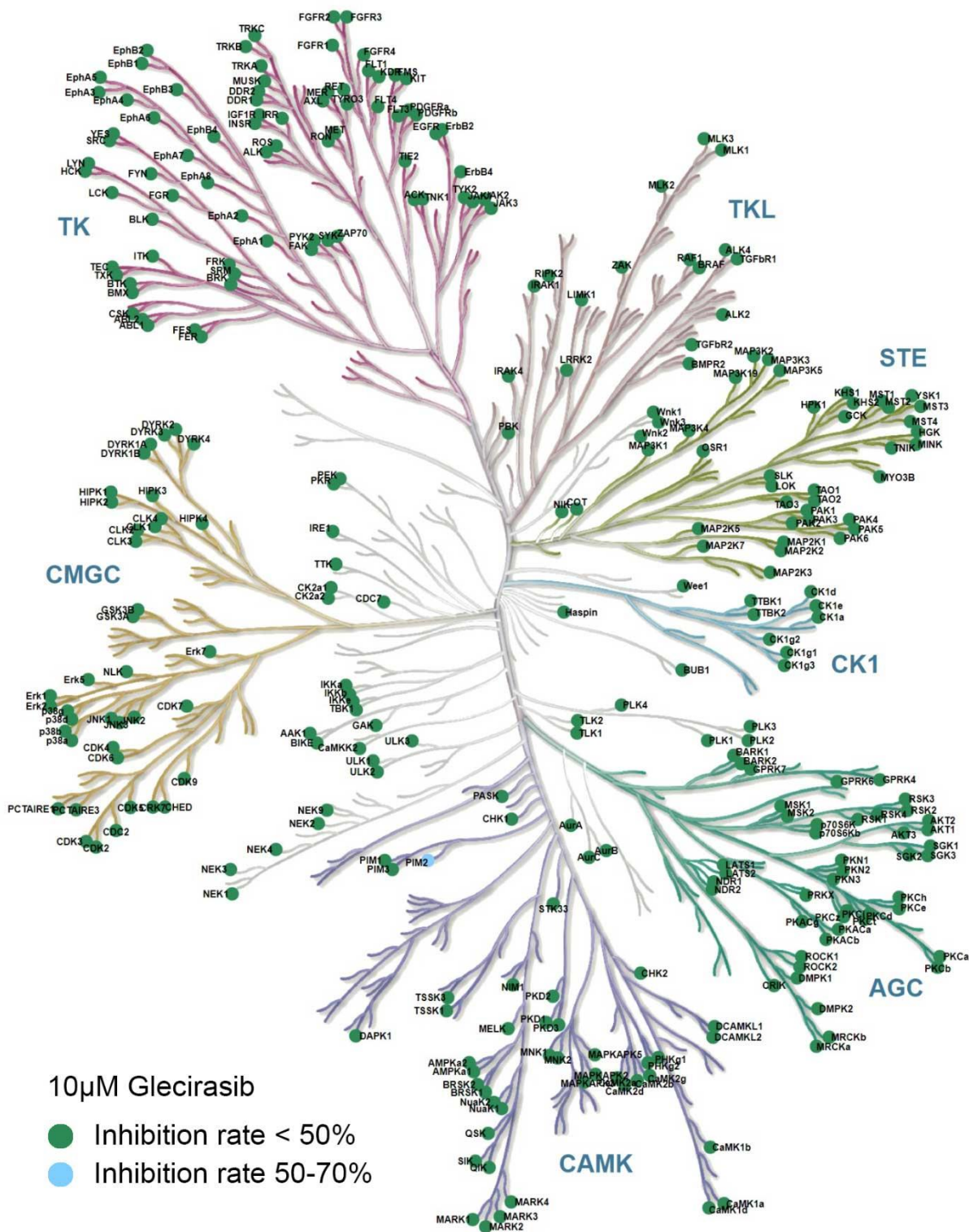

Supplement: Figure S1 — shows glecirasib's kinases profiling tree. [file crc-25-0001_figure_s1_suppsf1.pdf]
