# Supplementary material for: Glecirasib, a Potent and Selective Covalent KRAS G12C Inhibitor Exhibiting Synergism with Cetuximab or SHP2 Inhibitor JAB-3312
Source: Cancer Res Commun. 2025 May 14;5(5):792–803. doi: 10.1158/2767-9764.CRC-25-0001 (PMC12076188; doi:10.1158/2767-9764.CRC-25-0001)
Supplement: Table S1 — shows assay conditions of SOS1-mediated guanine nucleotide exchange assays. [file crc-25-0001_table_s1_suppst1.pdf]

Supplementary Table S1. Assay conditions of SOS1-mediated guanine nucleotide exchange assays.

| RAS Protein | RAS Conc. | Compound Pre-incubation | GDP Conc. | BODIPY ™ FL GTP | SOS1 ExD | MAb Anti 6HIS-Tb cryptate Gold | Exchange Incubation |
|---|---|---|---|---|---|---|---|
| GDP-KRAS G12D | 1.5 nM | 25 °C, 60 min | 5 nM | 80 nM | 0.5 μM | 52.5 ng/mL | 25°C, 4 h |
| GDP-KRAS G12V | 1.5 nM | 25°C, 60 min | 5 nM | 80 nM | 0.5 μM | 52.5 ng/mL | 25°C, 4 h |
| GDP-KRAS G12C | 3 nM | 25°C, 60 min | 5 nM | 80 nM | 0.5 μM | 52.5 ng/mL | 25°C, 4 h |
| GDP-KRAS WT | 0.4 nM | 25 °C, 60 min | 5 nM | 80 nM | 0.5 μM | 52.5 ng/mL | 25 °C, 4 h |
| GDP-NRAS WT | 1 nM | 25 °C, 60 min | 5 nM | 80 nM | 0.5 μM | 52.5 ng/mL | 25 °C, 3 h |
| GDP-HRAS WT | 1 nM | 25 °C, 60 min | 5 nM | 80 nM | 0.5 μM | 52.5 ng/mL | 25 °C, 2 h |

WT: wild type.
