# Supplementary material for: Glecirasib, a Potent and Selective Covalent KRAS G12C Inhibitor Exhibiting Synergism with Cetuximab or SHP2 Inhibitor JAB-3312
Source: Cancer Res Commun. 2025 May 14;5(5):792–803. doi: 10.1158/2767-9764.CRC-25-0001 (PMC12076188; doi:10.1158/2767-9764.CRC-25-0001)
Supplement: Table S2 — shows assay conditions of GppNp-RAS and cRAF interaction assays. [file crc-25-0001_table_s2_suppst2.pdf]

Supplementary Table S2. Assay conditions of GppNp-RAS and cRAF interaction assays.

RAS Protein	RAS Conc.	Compund Pre-incubation	cRAF RBD Conc.
GppNp-KRAS G12D	5 nM	25°C, 60 min	35 nM
GppNp-KRAS G12V	8 nM	25°C, 60 min	35 nM
GppNp-KRAS G12C	10 nM	25°C, 60 min	35 nM
GppNp-KRAS WT	5 nM	25°C, 60 min	50 nM
GppNp-NRAS WT	12 nM	25°C, 60 min	50 nM
GppNp-HRAS WT	10 nM	25°C, 60 min	50 nM

WT: wild type.
