# Supplementary material for: Glecirasib, a Potent and Selective Covalent KRAS G12C Inhibitor Exhibiting Synergism with Cetuximab or SHP2 Inhibitor JAB-3312
Source: Cancer Res Commun. 2025 May 14;5(5):792–803. doi: 10.1158/2767-9764.CRC-25-0001 (PMC12076188; doi:10.1158/2767-9764.CRC-25-0001)
Supplement: Table S3 — shows cell lines information of cellular assays. [file crc-25-0001_table_s3_suppst3.pdf]

Supplementary Table S3. Cell line information.

Cell Lines	Tissue of Origin	KRAS Alliterations	Cell Seeding Density, cells/well			
			2D CTG	3D CTG	p-ERK	p-AKT
NCI-H1373	Lung	KRAS p.G12C	-	1500	24000	-
NCI-H1792	Lung	KRAS p.G12C	2000	500	25000	-
NCI-H358	Lung	KRAS p.G12C	-	1500	40000	-
MIA PaCa-2	Pancreas	KRAS p.G12C	-	750	15000	-
SW1463	Colon	KRAS p.G12C	2000	1500	20000	20000
SW1573	Lung	KRAS p.G12C	2000	750	30000	-
SW837	Colon	KRAS p.G12C	3000	1500	30000	-
LS513	Colon	KRAS p.G12D	-	1500	50000	-
Capan-2	Pancreas	KRAS p.G12V	-	1500	30000	-
MKN-1	Stomach	KRAS WT AMP	-	1500	25000	-

WT: wild type; AMP: amplification; CTG: CellTiter-Glo.
