# Supplementary material for: Glecirasib, a Potent and Selective Covalent KRAS G12C Inhibitor Exhibiting Synergism with Cetuximab or SHP2 Inhibitor JAB-3312
Source: Cancer Res Commun. 2025 May 14;5(5):792–803. doi: 10.1158/2767-9764.CRC-25-0001 (PMC12076188; doi:10.1158/2767-9764.CRC-25-0001)
Supplement: Table S4 — shows kinases list in an order from strong to weak by inhibition rate of 10 μM glecirasib. [file crc-25-0001_table_s4_suppst4.pdf]

Supplementary Table S4. 330-kinase panel. Kinases are sorted from strong to weak by inhibition rate of 10 μM glecirasib.

No.	Kinase
1	PIM2
2	CaMK1β
3	HCK
4	AMPKα2/β1/γ1
5	AMPKα1/β1/γ1
6	PKCβ2
7	YES
8	AMPKα2/β2/γ2
9	TRKA
10	AKT2
11	EPHA5
12	RSK2
13	AurA
14	PAK1
15	EPHA3
16	TRKB
17	KIT
18	CHK2
19	MET
20	AMPKα2/β2/γ3
21	FYN [isoform b]
22	FGFR4
23	MER
24	PKD3
25	PHKG1
26	EPHB3
27	RSK1
28	EPHA4
29	PKD1
30	AMPKα2/β2/γ1
31	FGFR1
32	SGK
33	PIK3CB
34	PIM3
35	IKKβ
36	FAK
37	NEK2
38	CaMK2β
39	AKT3
40	RET
41	EPHB4
42	HER2
43	CaMK2δ
44	AMPKα1/β1/γ3
45	CaMK2α
46	TAOK2
47	FLT4
48	RSK3
49	RSK4
50	TSSK3
51	PRKCZ
52	PKD2

53	MAPKAPK2
54	HGK
55	p70S6K
56	PKR
57	AurC
58	AMPKα2/β1/γ2
59	PAK3
60	PYK2
61	PIM1
62	PHKG2
63	PKACα
64	AMPKα1/β2/γ3
65	DAPK1
66	AMPKα1/β1/γ2
67	NDR2
68	EGFR
69	PKACβ
70	SGK3
71	SYK
72	EPHA2
73	CDK6/CycD1
74	CHK1
75	EPHA8
76	AMPKα1/β2/γ2
77	Erk5
78	PRKCE
79	ITK
80	BUB1/BUB3
81	AKT1
82	TLK1
83	PLK3
84	SGK2
85	PKN3
86	PASK
87	NLK
88	WEE1
89	GPRK7
90	LRRK2
91	ACK
92	TSSK1
93	PIK3CD
94	TTBK1
95	BMP2K
96	ROCK2
97	TIE2
98	GLK
99	DDR1
100	TBK1
101	MYO3β
102	NIM1K
103	CDC7/ASK
104	CDK12/CycK
105	CSK
106	JNK1
107	PLK4
108	CDK4/CycD3
109	CDK4/CycD1

110	MARK1
111	CaMK2γ
112	FGFR3
113	IGF1R
114	MRCKα
115	NDR1
116	MLK1
117	MINK
118	CDK9/CycK
119	SRM
120	MST1
121	CDK1/CycA2
122	LIMK1
123	CDK13/CycK
124	TRKC
125	PRKCH
126	PAK2
127	PAK5
128	MAP3K3
129	ErK1
130	HIPK2
131	SIK
132	BLK
133	MAP3K19
134	CDK6/CycD3
135	PKN2
136	GPRK4
137	PKACγ
138	MAP2K7
139	CRIK
140	SLK
141	OSR1
142	AMPKα2/β1/γ3
143	SRC
144	PGK
145	ALK
146	CK2α1/β
147	p38δ
148	MNK2
149	WNK2
150	PAK6
151	ULK3
152	CDK1/CycB1
153	Nuak2
154	DMPK2
155	CLK2
156	PLK1
157	IKKα
158	EPHA1
159	MLK3
160	NEK4
161	JNK3
162	CRAF
163	CDK9/CycT2
164	PKCα
165	GSG2
166	PAK4

167	FES
168	MAP2K3
169	PRKX
170	PRKCQ
171	CLK3
172	MAP2K2
173	TGFβR1
174	CaMK1α
175	MST3
176	HIPK4
177	CDK5/p25NCK
178	LOK
179	BRK
180	MELK
181	P38α
182	DMPK1
183	LYNa
184	MSK2
185	ZAK
186	DDR2
187	MAP4K2
188	CK1α
189	DCAMKL2
190	KHS1
191	TAOK3
192	FLT1
193	CDK1/CycE1
194	STK33
195	MST2
196	p38β
197	GSK3α
198	ULK1
199	MAP3K1
200	ROCK1
201	PRKCD
202	CDK16/CycY
203	CAMKK2
204	HIPK1
205	TTK
206	CDK3/CycE1
207	NEK9
208	p38γ
209	CK1δ
210	GPRK6
211	DCAMKL1
212	CLK4
213	RIPK2
214	ALK2
215	TNK1
216	WNK3
217	BMPR2
218	COT
219	JAK1
220	DYRK4
221	RON
222	TYK2
223	LATS1

224	FER
225	BARK1
226	ROS
227	P70S6Kβ
228	NuaK1
229	AAK1
230	PBK
231	TAOK1
232	TLK2
233	MSK1
234	TNIK
235	MRCKβ
236	CDK2/CycA2
237	Erk7
238	BARK2
239	BRAF
240	DYRK1B
241	CK1ε
242	LATS2
243	PIK3CA
244	HIPK3
245	LYNb
246	CDK18/CycY
247	IRR
248	ABL2
249	MST4
250	MARK4
251	ULK2
252	EPHB1
253	TEC
254	CK2α2/β
255	MNK1
256	MARK2
257	MAP3K5
258	DYRK2
259	GSK3β
260	CDK9/CycT1
261	SIK3
262	ALK4
263	EPHB2
264	LCK
265	WNK1
266	MAP3K4
267	FGFR2
268	ABL1
269	DYRK1A
270	PEK
271	TTBK2
272	MAP3K2
273	AMPKα1/β2/γ1
274	KDR
275	CK1γ3
276	ERN1
277	MUSK
278	PKN1
279	CDK2/CycE1
280	MARK3

281	PRKCI
282	MAPKAPK3
283	MLK2
284	AurB
285	MAPKAPK5
286	BTK
287	PKCβ1
288	GAK
289	YSK1
290	CDK5/p35NCK
291	ZAP70
292	IRAK1
293	CLK1
294	CK1γ2
295	IRAK4
296	FYN [isoform a]
297	MAP2K5
298	PDGFRα
299	MAP2K1
300	JNK2
301	HER4
302	TGFβR2
303	FGR
304	INSR
305	NEK1
306	FRK
307	PLK2
308	CSF1R
309	CDK7/CCNH/MNAT1
310	AXL
311	JAK3
312	CaMK1δ
313	BRSK1
314	FLT3
315	JAK2
316	PDGFRβ
317	IKKε
318	QIK
319	CK1γ1
320	NEK3
321	BMX
322	Erk2
323	EPHA7
324	NIK
325	DYRK3
326	TYRO3
327	BRSK2
328	HPK1
329	TXK
330	EPHA6
