# Supplementary material for: Glecirasib, a Potent and Selective Covalent KRAS G12C Inhibitor Exhibiting Synergism with Cetuximab or SHP2 Inhibitor JAB-3312
Source: Cancer Res Commun. 2025 May 14;5(5):792–803. doi: 10.1158/2767-9764.CRC-25-0001 (PMC12076188; doi:10.1158/2767-9764.CRC-25-0001)
Supplement: Table S5 — shows IC50 values in SOS1 mediate nucleotide exchange assays. [file crc-25-0001_table_s5_suppst5.pdf]

Supplementary Table S5. IC_{50} values in SOS1 mediate nucleotide exchange assays.

GDP-bound RAS	IC_{50} (nM)		
	Glecirasib	Sotorasib	Adagrasib
GDP-bound KRAS G12C	2.28	5.87	0.618
GDP-bound KRAS G12D	>10,000	>10,000	9,444
GDP-bound KRAS G12V	>10,000	>10,000	>10,000
GDP-bound KRAS WT	>10,000	>10,000	3,873
GDP-bound HRAS WT	>10,000	>10,000	>10,000
GDP-bound NRAS WT	>10,000	>10,000	>10,000

WT: wild type.
