# Supplementary material for: Glecirasib, a Potent and Selective Covalent KRAS G12C Inhibitor Exhibiting Synergism with Cetuximab or SHP2 Inhibitor JAB-3312
Source: Cancer Res Commun. 2025 May 14;5(5):792–803. doi: 10.1158/2767-9764.CRC-25-0001 (PMC12076188; doi:10.1158/2767-9764.CRC-25-0001)
Supplement: Table S7 — shows IC50 values in p-ERK inhibition assays and cell viability assays. [file crc-25-0001_table_s7_suppst7.pdf]

Supplementary Table S7. IC_{50} values in p-ERK inhibition assays and cell viability assays.

Cell Line	Tissue of Origin	KRAS Alliterations	p-ERK IC_{50} (nM)			3D Cell Viability IC_{50} (nM)		
			Glecirasib	Sotorasib	Adagrasib	Glecirasib	Sotorasib	Adagrasib
NCI-H1373	Lung	p.G12C	6.61	27.5	26.9	5.03	5.22	4.58
NCI-H358	Lung	p.G12C	6.43	22.4	50.0	3.69	2.52	2.58
NCI-H1792	Lung	p.G12C	7.16	-	-	16.1	-	-
SW1573	Lung	p.G12C	14.0	61.8	101	138	-	-
SW837	Colon	p.G12C	13.6	-	-	11.8	-	-
SW1463	Colon	p.G12C	10.9	74.5	64.7	75.3	-	-
MIA PaCa-2	Pancreas	p.G12C	21.0	48.9	22.3	3.52	1.68	1.21
LS513	Colon	p.G12D	5,787	-	-	8,159	-	-
Capan-2	Pancreas	p.G12V	5,014	-	-	3,460	-	-
MKN-1	Stomach	WT AMP	8,384	-	-	10,000	>10,000	1,518

WT: wild type; AMP: amplification.
