# Supplementary material for: Glecirasib, a Potent and Selective Covalent KRAS G12C Inhibitor Exhibiting Synergism with Cetuximab or SHP2 Inhibitor JAB-3312
Source: Cancer Res Commun. 2025 May 14;5(5):792–803. doi: 10.1158/2767-9764.CRC-25-0001 (PMC12076188; doi:10.1158/2767-9764.CRC-25-0001)
Supplement: Table S8 — shows cell viability IC50 values in Ba/F3 engineered RAS cell lines. [file crc-25-0001_table_s8_suppst8.pdf]

Supplementary Table S8. Cell viability IC_{50} values in Ba/F3 engineered RAS cell lines.

**RAS mutation**	**IC_{50} (nM)**		
	**Glecirasib**	**Sotorasib**	**Adagrasib**
KRAS p.G12C	17.9	139	-
KRAS p.G12C/H95Q	6.43	47.3	-
KRAS p.G12C/H95D	24.3	134	-
KRAS p.G12C/Y96C	3,003	>10,000	-
KRAS p.G12C/Y96D	2,617	>10,000	-
KRAS p.G12C/R68S	68.6	663	-
KRAS p.G13C	6,095	>10,000	595
NRAS p.G12C	2.02	14.7	763
HRAS p.G12C	5.32	27.1	563
