# Supplementary material for: Glecirasib, a Potent and Selective Covalent KRAS G12C Inhibitor Exhibiting Synergism with Cetuximab or SHP2 Inhibitor JAB-3312
Source: Cancer Res Commun. 2025 May 14;5(5):792–803. doi: 10.1158/2767-9764.CRC-25-0001 (PMC12076188; doi:10.1158/2767-9764.CRC-25-0001)
Supplement: Table S9 — shows glecirasib's AUC in plasma and brain following a single oral dose. [file crc-25-0001_table_s9_suppst9.pdf]

Supplementary Table S9. Area under the curve (AUC) of glecirasib in plasma and brain following a single oral dose.

Species	Sex	Dose level (mg/kg)	Administration route	AUC_{0-24h}		Brain/Plasma AUC_{0-24h} ratio (%)
				Plasma (h·ng/mL)	Brain (h·ng/g)	
Mouse (NOD SCID)	Female	100	oral	25,696	955	3.72
Rat (Sprague-Dawley)	Male	300	oral	18,553	1,677	9.04
	Female	300	oral	18,420	1,875	10.2
