# Supplementary material for: Glecirasib, a Potent and Selective Covalent KRAS G12C Inhibitor Exhibiting Synergism with Cetuximab or SHP2 Inhibitor JAB-3312
Source: Cancer Res Commun. 2025 May 14;5(5):792–803. doi: 10.1158/2767-9764.CRC-25-0001 (PMC12076188; doi:10.1158/2767-9764.CRC-25-0001)
Supplement: Table S10 — shows the data of in vitro safety panel screening. [file crc-25-0001_table_s10_suppst10.pdf]

Supplementary Table S10. 41-target safety off-target activity panel.

Serial No.	Family	Target	Glecirasib (10 μM)	
			Mean % activation	Mean % inhibition
GPCR class				
1	Adenosine	A_{2A}	-1.49	4.28
2	Adrenergic	α_{1A}	27.61	22.91
3	Adrenergic	α_{2A}	22.71	27.95
4	Adrenergic	β_1	16.84	30.91
5	Adrenergic	β_2	6.84	45.33
6	Cannabinoid	CB_1	-2.83	-16.46
7	Cannabinoid	CB_2	-2.50	-3.75
8	Cholecystokinin	CCK_1	1.74	44.15
9	Dopamine	D_1	1.51	4.56
10	Dopamine	D_{2S}	-0.38	0.22
11	Endothelin	ET_A	1.59	-14.80
12	Histamine	H_1	1.79	16.22
13	Histamine	H_2	-0.43	1.71
14	Acetylcholine	M_1	13.78	31.00
15	Acetylcholine	M_2	3.59	22.51
16	Acetylcholine	M_3	45.97	18.32
17	Opioid	δ	8.42	32.99
18	Opioid	κ	10.20	20.93
19	Opioid	μ	5.69	36.87
20	5-Hydroxytryptamine	$5HT_{1A}$	0.27	29.29
21	5-Hydroxytryptamine	$5HT_{1B}$	7.33	-1.28
22	5-Hydroxytryptamine	$5HT_{2A}$	-0.04	13.63
23	5-Hydroxytryptamine	$5HT_{2B}$	2.59	21.70
24	5-Hydroxytryptamine	$5HT_{2C}$	19.92	43.20
25	Vasopressin	V_{1A}	2.09	9.46
Ion channel class				
26	Calcium channel	Cav1.2	NA	1.46
27	Sodium channel	Nav1.5	NA	1.01
28	Potassium channel	hERG	NA	18.22
29	Potassium channel	KCNQ1	NA	8.37
Transporter class				
30	Dopamine Transporter	DAT	NA	6.98
31	Norepinephrine Transporter	NET	NA	-35.69
32	Serotonin Transporter	SET	NA	4.04
Kinase class				
33	TK	LCK	NA	0.93
Enzyme class				
34	Cyclooxygenase	COX1	NA	32.33
35	Cyclooxygenase	COX2	NA	14.73
36	Cholinesterase	AChE	NA	-8.33
37	Phosphodiesterase	PDE3A	NA	12.50
38	Phosphodiesterase	PDE4D2	NA	10.29
39	Monoamine oxidase	MAO-A	NA	14.80
Nuclear Hormone Receptor class				
40	Androgen	AR	-33.00	114.81
41	Glucocorticoid	GR	-2.33	47.04
