# Supplementary material for: Glecirasib, a Potent and Selective Covalent KRAS G12C Inhibitor Exhibiting Synergism with Cetuximab or SHP2 Inhibitor JAB-3312
Source: Cancer Res Commun. 2025 May 14;5(5):792–803. doi: 10.1158/2767-9764.CRC-25-0001 (PMC12076188; doi:10.1158/2767-9764.CRC-25-0001)
Supplement: Table S11 — shows the summary of glecirasib's in vivo safety pharmacology studies. [file crc-25-0001_table_s11_suppst11.pdf]

Supplementary Table S11. *In vivo* safety pharmacology of glecirasib.

Organ Systems	Species/Strain	Gender/No. per Group	Noteworthy Findings	GLP Compliance
Cardiovascular System	Beagle Dog	3/gender/group	No effects on the lead-II ECG (heart rate, QRS duration, PR interval, RR interval, QT interval, corrected QT interval) and blood pressure were noted in conscious and unrestrained Beagle dogs following a single oral dose of glecirasib at 50, 150 and 450 mg/kg.	Yes
Respiratory system	Sprague Dawley Rat	5/gender/group	No remarkable effect on respiratory functions was induced in conscious and unrestrained rats following a single oral dose of glecirasib at 100, 300 and 1000 mg/kg.	Yes
Central Nervous System	Sprague Dawley Rat	5/gender/group	No effects on neurobehavioral performances were induced in conscious and unrestrained rats following a single oral dose of glecirasib at 100, 300 and 1000 mg/kg.	Yes
