# Supplementary material for: Glecirasib, a Potent and Selective Covalent KRAS G12C Inhibitor Exhibiting Synergism with Cetuximab or SHP2 Inhibitor JAB-3312
Source: Cancer Res Commun. 2025 May 14;5(5):792–803. doi: 10.1158/2767-9764.CRC-25-0001 (PMC12076188; doi:10.1158/2767-9764.CRC-25-0001)
Supplement: Table S12 — shows the summary of glecirasib's genotoxicity and phototoxicity studies. [file crc-25-0001_table_s12_suppst12.pdf]

Supplementary Table S12. Genotoxicity and phototoxicity of glecirasib.

Study	Species/Strain	Gender/No. per Group	Noteworthy Findings	GLP Compliance
In vitro Ames	Five histidine auxotroph strains of S. typhimurium: TA97a, TA98, TA100, TA102 and TA1535	NA	No mutagenic effects were induced in all test strains by glecirasib at 50, 150, 500, 1500 and 5000 μg/plate in the presence or absence of S9 mix. The result of this Ames test was concluded to be negative.	Yes
In vitro mammalian chromosome aberration test	Chinese Hamster Lung (CHL) cells	NA	No significant increase was observed in chromosome structural aberration rate of CHL cells exposed to glecirasib at 7.5, 15, 30 and 45 μg/mL for approximately 4 hr in the presence or absence of S9 mix; no significant increase was observed in chromosome structural aberration rate of CHL cells exposed to glecirasib at 3.75, 7.5 and 15 μg/mL for approximately 24 hr in the absence of S9 mix. The result of this *in vitro* CHL cells chromosome aberration test was concluded to be negative.	Yes
Rat bone marrow micronucleus assay	Sprague Dawley Rat	5/gender/group	No damage on chromosomal integrity or abnormal chromosome segregation in the bone marrow was noted after SD rats were administered with glecirasib at 200, 600 and 2000 mg/kg via oral gavage once daily for 3 consecutive days. The result of this micronucleus assay was negative.	Yes
Phototoxicity Study	England Guinea Pig	4/gender/group	No phototoxicity was observed when England guinea pigs were administered with a single dose of glecirasib at 300 and 1000 mg/kg via oral gavage followed by UV irradiation on the skin.	Yes
